# Supplementary material for: NIPAm-Based Modification of Poly(L-lysine): A pH-Dependent LCST-Type Thermo-Responsive Biodegradable Polymer
Source: Polymers (Basel). 2022 Feb 18;14(4):802. doi: 10.3390/polym14040802 (PMC8962975; doi:10.3390/polym14040802)
Supplement: Supplementary file 1 [file polymers-14-00802-s001.zip › polymers-1583169-supplementary.pdf]

NIPAm-based Modification of Poly(L-lysine): A pH-dependent LCST-type Thermoresponsive biodegradable polymer.

Aggeliki Stamou,[a] Hermis Iatrou,[b] Constantinos Tsitsilianis [a*]

[a] *Department of Chemical Engineering, University of Patras, 26500 Patras, Greece*

[b] *Department of Chemistry, University of Athens, Panepistimiopolis, Zografou, 15771 Athens, Greece*

Synthesis of PLL. *E-tert*-Butyloxycarbonyl-L-lysine-NCA was synthesized firstly and used as the protected α-amino acid *N*-Carboxyanhydride monomer for the ring opening polymerization to produce *poly(N^{ε}-Boc-L-lysine)*, according to standard methods.[1]

Synthesis of ε-tert-butyloxycarbonyl-L-lysine N-carboxy anhydride (BOC-LL-NCA). Briefly, N^{α}, N^{ε}-Di-(*tert*-butoxycarbonyl)-L-lysine was added into a flask, placed on the vacuum line, and pumped overnight. Then purified ethyl acetate was distilled, followed by argon insertion to reach atmospheric pressure and by addition of triphosgene. The mixture was left to react for 10 minutes. Triethylamine diluted in dry ethyl acetate was subsequently added dropwise, and the solution was immersed in an ice-water bath for 6 hours. The precipitate was filtered, in order to remove the HCl salt of triethylamine, the clear solution was immersed in an ice bath, and the NCA was extracted with Milli-Q water repeatedly, until neutral pH of the aqueous phase was achieved. The purified NCA was recrystallized three times under high vacuum in a custom-made apparatus, with ethyl acetate/hexane (1/5 v/v) pair at –20 °C. The purity was confirmed by ^{1}H NMR.

Synthesis of poly(N^{ε}-Boc-L-lysine). The polymerization was performed in a glass custom-made apparatus equipped with a high vacuum stopcock for periodic degassing of the solution and a magnetic stir bar covered with glass. The apparatus was initially attached to the vacuum line through a ground joint and was evacuated and flame dried several times, followed by distillation of 50 mL of highly pure DMF into the main flask. The stopcock was closed and the apparatus was then inserted in the glove box to add the appropriate amount of *ε-tert*-butyloxycarbonyl-L-lysine-NCA in a side ampoule with a second stopcock. Then, the apparatus was attached again at the vacuum line and 5 mls of purified DMF was distilled into the ampoule containing the NCA, followed by heat sealing of the glass to form an ampoule of a solution of the NCA. Then, an ampoule of the dimethylamine was ruptured and was added at the round bottom flask of the

reactor, followed by the addition of the amount of the NCA by rupturing the corresponding ampoule. The consumption of the monomers was monitored by FT-IR through removal of an aliquot of the solution in the glove box. Periodically, the solution was pumped to remove the CO_2 produced from polymerization. After completion of the polymerization, the polymer was precipitated in diethylether and dried under high vacuum. The poly(N^{ε}-Boc-L-lysine) synthesis was confirmed by ^{1}H NMR.

Gel Permeation Chromatography (GPC) was performed for the molecular characterization of protected polypeptide. The system was composed of a Waters 600 HPLC pump, Waters, Ultrastyragel columns (HT-2, HT-4, HT-5E, and HT-6E), a Waters 410 differential refractometer, and a Precision PD 2020 two angles (15°, 90°) light scattering detector at 60 ^{o}C. The carrier solvent used was a solution of 0.1 M LiBr in DMF with a flow rate of 1 mL min^{-1}.

Deprotection of poly(N^{ε}-Boc-L-lysine). The deprotection procedure was carried out as follows. 0.77g of poly(N^{ε}-Boc-L-lysine) and 10 mL of dichloromethane (CH_2Cl_2, DCM) were added in a 50 mL round-bottomed flask. The solution was left under vigorously stirring for 30 minutes to dissolve in CH_2Cl_2 which is a good solvent for the protected polypepride. Then, 4 mL of trifluoroacetic acid (CF_3COOH, TFA, ≥99.5%) were added to the solution and left to react under stirring for two hours at room temperature. Accordingly, the reaction flask was fitted to a rotary evaporator, for the removal of DCM and TFA. The remaining polymer was left to dry for two days. Then, the polymer was dissolved in water and the resulted solution was neutralized by the addition of potassium carbonate (K_2CO_3), until the pH reached a neutral value. The deprotected polypeptide was purified by dialysis using a membrane of MWCO = 3.5 kDa. The dialysis process lasted several days and finally a white solid polymer was obtained by freeze-drying. After drying, the deprotected poly-L-lysine (PLL_{50}) was stored in the refrigerator. The deprotection of PLL was quantitative as confirmed by ^{1}H-NMR since the proton peaks of the boc group ($-CH_3$) at 1.1 ppm disappeared.

Characterization of PLL-g-NIPAm samples. The modified PLL-g-NIPAm were characterized by ^{1}H NMR in deuterated water (D_2O). A characteristic ^{1}H-NMR spectra is presented in Figure S1.

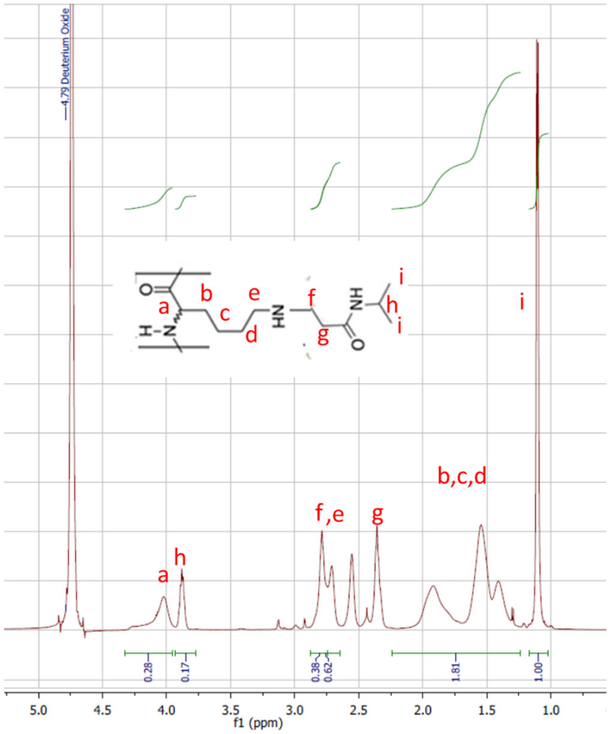

Figure S1. Example of ^{1}H-NMR spectrum of PLL-g-NIPAmX

Figure S1 displays the ^{1}H-NMR spectra of a modified PLL-g-NIPAm in D_2O: the peak at δ 4.8 ppm corresponds to the peak of the deuterated solvent, D_20, 1.2-2 ppm corresponds to proton of b, c, d,-CH_2 groups of PLL, ~ 2.7 ppm corresponds to the protons of PLL e-CH_2 next to the primary amino group, 4.1 ppm corresponds to the proton of α-CH of the PLL backbone. At 3.8-3.9 ppm the h proton, CH, of the isopropyl group, 2,3 ppm the g protons of -CH_2-CO-, 2.9-3.0 ppm the f protons next to the secondary NH and 1.0 ppm the six protons of the isopropyl group (i) of NIPAm respectively. Unfortunately, we are not able to discriminate the mono and di-adduct addition. The mole % of NIPAM conjugation was determined by the ratio of the integrated peaks h/a or i/a ([NIPAm] x 100/[LL]).

References

[1] Hadjichristidis, N.; Iatrou, H.; Pitsikalis, M.; G Sakellariou, G. Synthesis of Well-Defined Polypeptide-Based Materials via the Ring-Opening Polymerization of α-Amino Acid *N*-Carboxyanhydrides. *Chemical reviews* **2009**, *109* (11), 5528-5578.
